# Recent advances in treatments for resynchronization of ovulation in small ruminants: a review

**DOI:** 10.1590/1984-3143-AR-2022-011

**Published:** 2023-04-14

**Authors:** Isabel Oliveira Cosentino, Mário Felipe Alvarez Balaro, Alejo Menchaca, Raquel Perez-Clariget, Rodolfo Ungerfeld, Felipe Zandonadi Brandão

**Affiliations:** 1 Faculdade de Veterinária, Universidade Federal Fluminense, Niterói, RJ, Brasil; 2 Instituto de Reproducción Animal Uruguay, Fundación IRAUy, Montevideo, Uruguay; 3 Plataforma de Investigación en Salud Animal, Instituto Nacional de Investigación Agropecuaria, Montevideo, Uruguay; 4 Departamento de Producción Animal y Pasturas, Facultad de Agronomía, Universidad de la República, Montevideo, Uruguay; 5 Departamento de Biociencias Veterinarias, Facultad de Veterinaria, Universidad de la República, Montevideo, Uruguay

**Keywords:** estrous synchronization, estrous induction, ewes, does, hormonal protocols

## Abstract

Hormonal methodologies to control small ruminants’ estrous cycle are worldwide used and evolved, adjusting the application to the precise female physiological moments to enhance reproductive performance. The estrous cycle can be induced and/or synchronized, aiming for fixed-time artificial insemination, or based on estrus behavior signs for insemination, natural or guided mating. Successive protocols can be performed to resynchronize ovulation and increase reproductive outcomes in females that failed to conceive. These recently developed treatments aim to resynchronize the ovulation as earlier as non-pregnancy is detected. The present review aimed to summarize the recent advances and main findings regarding resynchronization protocols used in small ruminants. Lastly, we present future perspectives and new paths to be studied in the subject. The resynchronization treatment is still a growing field in small ruminant reproduction, nevertheless, some enhancements are found in the reproductive outcome, showing that such protocols can be successfully used in sheep and goat production.

## Introduction

Although research in techniques for handling the estrous cycles began more than 70 years ago, protocols more frequently used for estrous synchronization in small ruminants still provide variable results. The studies directed to understand the physiology of the estrous cycle in small ruminants started during the1920s (Drummond-Robinson and Asdell, 1926), but the pharmacological attempts to control the reproductive physiology began later, in the 1940s. Initially, the studies began with the use of gonadotrophins for the induction of estrus in anestrous ewes and goat does (Van Der Noot et al., 1946; Folley et al., 1949), the subsequent studies tried to mimic the luteal phase with progestogens (Dutt and Casida, 1948; O’mary et al., 1950), and later studies aimed to induce the luteolysis with prostaglandins (Thorburn and Nicol, 1971; Douglas and Ginther, 1973). In small ruminants, it is important to carefully consider the physiology of each species and breed, as the degrees of reproductive seasonality vary widely, thus, while some breeds present pronounced seasonality, others are almost non-seasonal (Gómez-Brunet et al., 2011; Ramírez et al., 2021). Therefore, hormonal protocols can be used for estrous synchronization (during the breeding season) or induction of synchronized estrus (outbreeding season, during the postpartum or before puberty).

One of the major advances in reproductive technologies in livestock in the last years has been the new protocols for Fixed Time Artificial Insemination (FTAI). From the understanding of follicular dynamics in the 1990s and 2000s, novel strategies were developed to improve the implementation of FTAI, in cattle (Mapletoft et al., 2018) as in sheep and goats (Menchaca and Rubianes, 2004). To minimize the incidence of non-productive intervals when FTAI programs are implemented, successive protocols are required in unpregnant females, resynchronizing their ovulation during the shortest period possible (Cosentino et al., 2019). This requires a precise diagnosis of non-pregnant females as early as possible to apply a second treatment, or the application of blind treatments to all females ensuring that these treatments do not affect the establishment of pregnancies or the early embryo development in pregnant females.

In this sense, this review aimed to update and summarize the existing knowledge on hormonal treatments used for resynchronization of ovulation, especially in sheep and goats. Hence, this review highlights the main concerns regarding the protocols, as well as the steps for performing them, it is, the main drugs used, the timing and evaluations done during the protocol. Finally, although the state of the art is still limited, the review presents the main studies related to the resynchronization protocols in small ruminants and highlights the advantages/disadvantages of the on-farm application of these protocols.

## General aspects

Hormonal protocols are widely used to manipulate small ruminants’ estrous cycles. Progestogens are usually administered using intravaginal devices, including silicone-based devices impregnated with progesterone (P4), such as Controlled Internal Drug Release (CIDR) and Dispositivo Intravaginal Caprino Ovino (DICO) (Wheaton et al., 1993; Vilariño et al., 2010), or, intravaginal sponges (IVS) impregnated with progesterone analogues, as medroxyprogesterone acetate (MAP) or flurogestone acetate (FGA) (Barrett et al., 2008; Santos et al., 2010). There are some preliminary studies on the administration of long-acting injectable progesterone (Rodríguez-Martínez et al., 2018; Alvarado-Espino et al., 2019; Amaral D’Avila et al., 2022; Payan et al., 2022), but the results are still inconsistent. Until now, sponges impregnated with MAP have been used for the resynchronization protocol (Miranda et al., 2018; Cosentino et al., 2019, 2021, 2022), but to the best of our knowledge, there are no studies with other progestogens.

An important risk for resynchronization protocols before pregnancy diagnosis, was the possible negative effect of the insertion of a progestogen device on the functionality of the endogenous corpus luteum, and therefore, possible fetal loss. In this sense, even though the increase in serum P4 levels induced by CIDR-type treatments administered early in the luteal phase (i.e., 0 to 3 days after ovulation) could have a positive effect on the uterine environment and conceptus development (Pugliesi et al., 2014), other studies reported that it can affect the luteal function. In effect, Menchaca and Rubianes (2001) and Pugliesi et al. (2014) reported that the insertion of a novel device in some conditions can affect the functionality of the corpus luteum in goats and cattle, respectively. Discarding this risk is essential, as for early resynchronization protocols in small ruminants, the second device should be inserted later in the luteal phase (e.g., 12 days after ovulation).

In sheep, protocols based on synthetic progestogens led to a reduction of the endogenous P4 serum values but did not affect pregnancy nor trigger luteolysis if the devices are inserted 12 d after ovulation (Miranda et al., 2018). Consistently, Cosentino et al., (2019) corroborated that the insertion of a second MAP device 12 d after ovulation does not affect the endogenous P4 serum concentration despite whether ewes are pregnant or not. In goats, a first attempt performed in a commercial flock showed that similarly to sheep, the second progesterone device did not interfere with the gestational CL (Cosentino et al., 2020). In these studies, the insertion of a second MAP sponge did not affect the luteal blood flow nor the P4 production during the following days, and these results did not differ in pregnant or non-pregnant females. Therefore, the proposed protocols are proven to be secure for application in females in which pregnancies were still not diagnosed.

## Resynchronization of ovulation

Resynchronization protocols have been developed to shorten the females' unproductive time, mainly when it is intended to carry out sequential FTAI in the same females. These treatments have been successfully developed in cattle since the beginning of the century (Stevenson et al., 2003; Sani et al., 2011), once the FTAI technology was implemented in that species. Therefore, treatments in small ruminants are based on what has been previously proposed in cattle. In general, resynchronization protocols in cattle repeat similar treatments to those previously used for FTAI, over-imposed during the following luteal phase. Treatments used for resynchronization of the ovulation without knowing pregnancy status should not include prostaglandin analogues as this would induce luteal regression, and thus, pregnancy loss. To minimize those risks, beef cattle protocols are mainly based on GnRH analogues or estradiol (Bó et al., 2016).

If prostaglandins are to be used in the resynchronization protocol, this should be done after it has been confirmed that the female is not pregnant. This is of practical importance, as the inclusion of these analogues is useful to synchronize better the luteolysis and indirectly the ovulation, thus an alternative recently developed is the early non-pregnancy diagnosis based on the luteal blood flow. Although this technique does not allow discarding the occurrence of premature luteal regressions, provides a great accuracy on that corpus luteum that effectively, are not functional, and therefore, the administration of prostaglandin analogues would not be a problem. The early diagnosis of non-pregnancy allows the administration of prostaglandin analogues as a component of the resynchronization protocols, without affecting the conception rate when compared to the use of other hormones (Sani et al., 2011). Other protocols, based on the application of eCG at the time of removal of the P4 device, promote the growth of the dominant follicle, and, consequently, an increase in the ovulation rate, the ovulation of larger follicles with greater production of endogenous estradiol, better oocyte competence, greater luteal diameter and, therefore, a greater P4 values in the following luteal phase (Bó et al., 2016).

Originally, resynchronization treatments in cattle were used just after the lack of an identification of the conceptus in the uterine environment (Pereira et al., 2013). However, recently, these protocols included an early non-pregnancy diagnosis based on luteal blood flow evaluation (Correa-Calderón et al., 2016), which allows applying resynchronization treatments as early as 22 to 28 days after the first artificial insemination (AI) (Sinedino et al., 2014; Pessoa et al., 2018), or even earlier to allow FTAI at intervals like the natural estrous cycle (Pugliesi et al., 2019; Palhão et al., 2020). This requires that the second progestogen should be inserted between 14 and 21 days after the first AI, without harming early pregnancy as it cannot be diagnosed by ultrasound so early.

### Treatments in sheep

In sheep, initially, Miranda et al. (2018) presented a protocol that consisted of a traditional estrus synchronization treatment followed by AI or natural mating. The resynchronization treatment includes the insertion of a new P4 intravaginal device fourteen days later, remaining *in situ* for six days. After its removal, all ewes were submitted once again to AI or NM without a previous pregnancy diagnosis, thus, even being pregnant. At the second service, after the resynchronization protocol, ewes from each group showed similar pregnancy rates, also, despite not being subjected to an early non-pregnancy diagnosis before the resynchronization treatment, premature embryo loss was not detected, indicating that resynchronization of the ovulation can be effectively combined with FTAI, and can be used, even in already pregnant females. However, this treatment implied that all ewes should be retreated, involving great costs of hormonal treatments, losing semen doses in pregnant ewes, increasing the environmental negative impacts, and requiring extra handling during animals' early pregnancy, with the increase in the risk of pregnancy losses. Therefore, like what was reported in cattle, Arashiro et al. (2018) validated in sheep the use of US doppler scan of the ovaries for non-pregnancy diagnosis 17 days after FTAI, and consequently allowing the use of resynchronization treatment only in non-pregnant ewes. This diagnosis is based on the study of the corpus luteum blood perfusion by US doppler, and in sheep allows to effectively determine which females are not pregnant as early as 17 days after ovulation (Arashiro et al., 2018).

**Figure 1:**
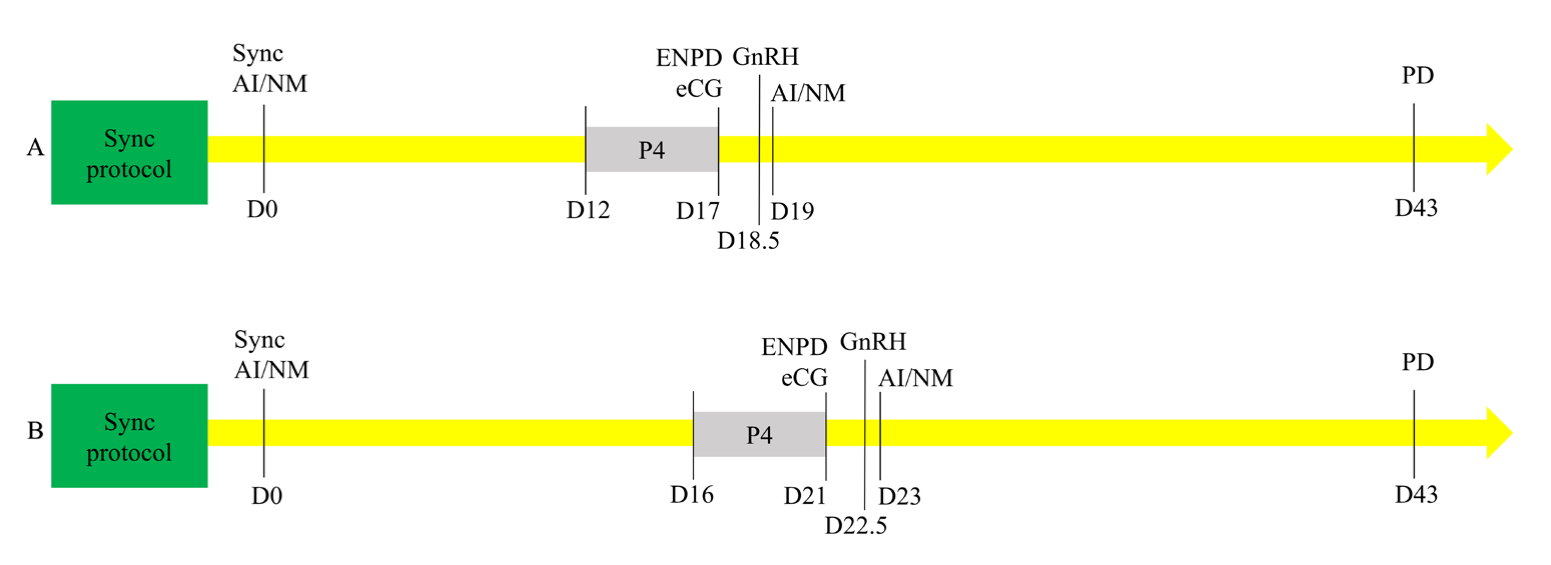
A) Resynchronization protocol for ewes adapted from Cosentino et al. (2019); B) Resynchronization protocol for does adapted from Cosentino et al. (2022). D – Day; eCG – equine Chorionic Gonadotrophin; GnRH – Gonadotrophin Releasing Hormone; P4 – progestogen device; AI – artificial insemination; NM – natural mating; ENPD – early non-pregnancy diagnosis (luteal evaluation); PD – pregnancy diagnosis (uterine/fetal evaluation).

Cosentino et al. (2019) also proposed a protocol using a lower dose of eCG for the resynchronization of ovulations, as the use of 200 IU synchronized better the second ovulation than 300 IU, the dose used for the initial FTAI in ewes. Thus, with this information, treatments were combined, beginning with the insertion of an IVS impregnated with MAP for six days, and one day before sponge removal, females received 300 IU of eCG and 0.24 mg of cloprostenol sodium. Finally, 24 h after sponge withdrawal, ewes received 0.025 mg of lecirelin acetate (GnRH analogue) and were inseminated 27 – 30 h after lecirelin administration with fresh semen (Figure 1A). Twelve days later, a new IVS was inserted, remaining in situ for five days, when the early non-pregnancy diagnosis was performed, allowing to separate those ewes that had a functional or a non-functional corpus luteum. Immediately after, the IVS was removed, 200 IU of eCG were administrated, followed by the administration of a GnRH analogue 36 h later. The second AI was performed 9 – 12 h later, and the final pregnancy diagnosis was performed 40 days later, 59 days after the initial AI (Cosentino et al., 2019). Results showed an increase from 19.4% of pregnant ewes in the first AI to 41.9% in the second with 47.8% of accumulated confirmed pregnancy.

This treatment was tested also in different categories of sheep (Cosentino et al., 2021) and nulliparous ewes can respond similarly to multiparous ewes to resynchronization. However, the response of early postpartum ewes during the breeding season was strongly affected resulting in very low outcomes. In these studies, the protocol used was adapted from Cosentino et al. (2019), only delaying the time of the first GnRH administration (36 h after the IVS removal). By comparing the results of females that did and did not receive the second protocol, it was confirmed that the resynchronization protocol did not affect results from the first FTAI by inducing premature luteal regression or fetal losses (Cosentino et al., 2021).

### Treatments in goats

In general, these treatments are less advanced in goats. Similarly to what was reported in cattle and sheep, it was demonstrated in goats that the evaluation of luteal blood flow for non-pregnancy diagnosis can be performed as early as 21 days after FTAI (Cosentino et al., 2018), and that the insertion of a second progesterone device did not interfere with the gestational corpus luteum (Cosentino et al., 2020). Therefore, it was proposed a resynchronization protocol (Figure 1B) in which the does were synchronized by the insertion of a MAP containing IVS for six days (Day -8 to -2), followed by the administration of 200 IU of eCG and 0.12 mg of cloprostenol sodium one day before sponge withdrawal (Day -3). Thirty-four hours after the sponge removal, does received a GnRH analogue (Day -1), and the FTAI should be performed at Day 0. Sixteen days later, a new IVS was inserted, remaining *in situ* for five days, when early non-pregnancy diagnosis should be performed according to luteal blood flow (Day 21). At IVS removal, the use of saline or 100 IU of eCG was compared, with no administration of GnRH analogue. Also, the breeding management was assessed: natural mating was performed from the sponge removal for 4 days, and AI was performed following estrous behavior and ovulation detection. There were no differences in using or not of eCG in pregnancy outcomes (Cosentino et al., 2022). Since the first synchronization was not bred, in the present review we highlight the suggested moments for FTAI and early non-pregnancy diagnosis, in a complete protocol yet to be studied.

## Perspectives

**Figure 2:**
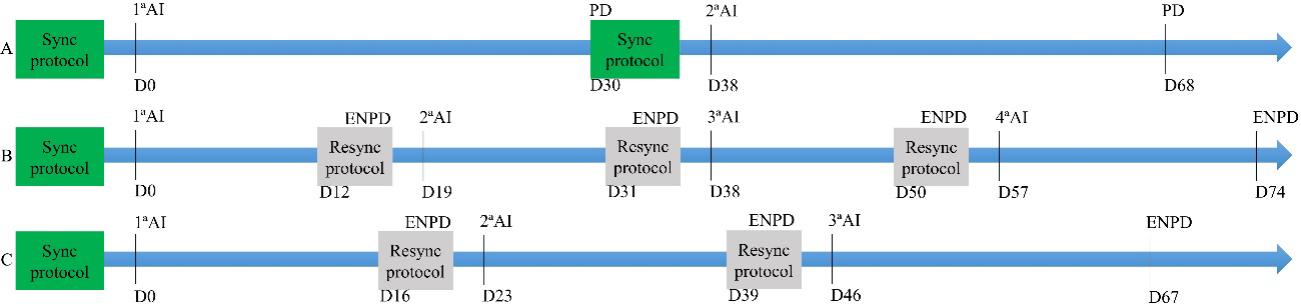
A) 75 days traditional breeding season calendar. B) Proposed successive resynchronizations for ewes. C) Proposed successive resynchronizations for goats. D – Day; AI – artificial insemination; ENPD – early non-pregnancy diagnosis (luteal evaluation); PD – pregnancy diagnosis (uterine/fetal evaluation).

In sheep and goats, resynchronization protocols appear as a promising tool to improve females’ reproductive performance after FTAI is implemented in these species. Besides reducing the unproductive time of a ewe/doe in the flock and granting new chances of becoming pregnant, resynchronization protocols associated with the use of early diagnosis of the non-pregnant female allow the reduction of using unnecessary hormones, (2) prevention of the semen doses loss, enabling investment in more expensive breeders, and (3) improvement of overall results of reproductive programs by applied sequential FTAI, shortening the total work period and simplifying practices. In addition, females early detected as pregnant can have nutritional management balanced promptly, in addition to avoiding unnecessary animal handling during early pregnancy, decreasing the risks of early pregnancy losses. Also, even though not yet studied, successive resynchronization treatments enhance the number of chances given for a female to get pregnant compared to traditional protocols for FTAI that require waiting for pregnancy diagnosis around 30 days after insemination (Figure 2).

However, it should be considered that protocols for resynchronization of ovulation in small ruminants are still being developed. More attempts should be performed to improve reproductive indices and identify the best treatments to enhance each category's outcomes. In sheep, studies are a bit more advanced, even though such techniques should be tested in different breeds, climates, and different reproductive statuses (multiparous, nulliparous, primiparous, early post-partum, lactating, and seasonal anestrus) and with different synchronization protocols (progestogens vs prostaglandin-based protocol, and different sources of progestogens). In goats, a preliminary protocol should be still settled. Considering results obtained until the present moment, a protocol like that used in ewes could be applied to goats, whether considering the similarities and differences between the species. The association of treatments for resynchronization of the ovulation with early pregnancy diagnosis allows for reducing the use of unnecessary hormones, avoids the loss of sperm doses, and shortens the lambing/kidding interval, which concentrates births and homogenizes the lambs/kids batch. Also, more opportunities to impregnate are given (two FTAI within about 20 days), with the aid of the identification of early pregnant ewes/does prevent unnecessary management and implementing adequate nutrition management of the pregnant females.
